# A design for life: Predicting cognitive performance from lifestyle choices

**DOI:** 10.1371/journal.pone.0298899

**Published:** 2024-04-16

**Authors:** Emily S. Nichols, Georgia Nelson, Conor J. Wild, Adrian M. Owen

**Affiliations:** 1 Applied Psychology, Faculty of Education, Western University, London, Ontario, Canada; 2 Western Institute for Neuroscience, Western University, London, Ontario, Canada; 3 Neuroscience Program, Schulich School of Medicine and Dentistry, Western University, London, Ontario, Canada; 4 Department of Physiology and Pharmacology, Western University, London, Ontario, Canada; 5 Department of Psychology, Western University, London, Ontario, Canada; The University of Adelaide, AUSTRALIA

## Abstract

Maintaining cognitive capacity through adulthood has been the target of many recent studies that have examined the influence of lifestyle choices such as exercise, diet, and sleeping habits. Many of these studies have focused on a single factor (e.g., diet) and its effect on cognitive abilities; however, humans make numerous lifestyle choices every single day, many of which interact and influence each other. Here, we investigated whether combinations of lifestyle choices can predict better or worse cognitive performance in the general population, and whether optimal combinations of choices existed depending on the cognitive domain. Specifically, we examined 20 self-reported lifestyle choices, such as playing video games, drinking alcohol, and amount of exercise taken, in a sample of almost 10,000 participants. All participants also completed 12 cognitive tests that have been shown to generate three composite cognitive domain scores pertaining to short-term memory, verbal abilities, and reasoning. Using recursive feature elimination and random forest regression, we were able to explain 9% of the variance in short-term memory scores, 8% of the variance in reasoning scores, and 7% of the variance in verbal ability scores. While the regression model provided predictive power in all three domains, these levels indicate that even when considering a large number of lifestyle choices, there remains a considerable degree of variability in predicting short-term memory, reasoning and verbal abilities. Thus, while some modifiable lifestyle factors may have an impact on cognitive capacity, there likely exists no single optimal design for life.

## Introduction

There is a strong incentive to improve our cognitive abilities; factors such as vocational success, levels of happiness, and even life expectancy are all linked to cognitive health [1–4]. A natural extension of any discussion about cognitive health is the relevance of lifestyle choices to differences in cognitive abilities; that is, whether some modifiable behavioural practices affect cognitive health more than others, and whether it is possible to optimize one’s lifestyle choices to maximize cognitive advantages [5, 6]. Unsurprisingly therefore, maintaining cognitive capacity throughout life has been the target of many recent studies that have examined the influence of lifestyle choices such as exercise, diet, and sleeping habits. In parallel, a vibrant industry has emerged, focusing on the maintenance of cognitive health. Complicating matters, however, is that cognitive health is remarkably heterogeneous across individuals [7, 8].

The relationship between cognitive health and some lifestyle choices is already well documented in the literature. For example, adults of all ages who regularly sleep for between 7–8 hours per night perform better on some aspects of cognition than those who sleep less, or more, than that amount [9, 10]. Older adults who sleep longer also tend to perform more poorly on the Mini Mental State Examination (MMSE) and other measures of cognitive function [11, 12]. Aerobic training and physical activity have also been associated with better cognition and a lower risk of cognitive impairment in multiple studies [13–15]. Finally, regular social contact has been consistently associated with better cognitive ability in older adults [16–18]. For example, in one 30-year longitudinal study, loneliness was found to be associated with increased cognitive decline and lower baseline cognitive abilities, while married individuals deteriorated less quickly than those who were single [18].

Lifestyle choices that may affect cognitive health also include hobbies and other purely recreational activities, although there exists considerable disagreement in the literature. For example, Green and Bavelier (2003) [19] reported that playing action-based video games markedly improved visual selective attention. Similarly, Basak and colleagues (2008) [20] reported a positive correlation between video game training and improvements in tests of visual selective attention, task switching, visual short-term memory, reasoning and working memory. In contrast, however, several studies have shown that improvements in cognitive abilities following video game training do not generalize beyond the tasks that were specifically trained [21–23]. Similarly, despite the many claims made about the general cognitive benefits of ‘brain training’ [24, 25], numerous studies have found that improvements made on the cognitive tasks that were trained do not transfer to untrained tasks [26–30].

Many of the studies reviewed above have focused on how a single lifestyle choice may affect cognitive health, yet humans make many different lifestyle choices every day. In one longitudinal study that examined several factors simultaneously, vigorous exercise, volunteerism, and non-smoking were all related to good cognitive health in elderly participants [5]. In another cross-sectional study of 2,315 cognitively healthy older adults, physical activity, a healthy diet, cognitive and social activity, and light-to-moderate alcohol consumption were positively associated with cognitive function [6]. Finally, meta-analyses in older participants have routinely identified positive relationships between cognition and social engagement, physical activity, and therapeutic nutrition [31–33], although little is known about how such factors influence cognition in younger groups.

One way of examining complex relationships between many predictors is through machine-learning. Random forest regression is one such machine learning procedure, in which many decision trees (that is, models that use binary splits on predictor variables to produce outcome predictions) are constructed and aggregated to give a prediction for each observation [34]. Random forest regression is particularly useful in this regard, especially when predictors may not have a linear relationship with cognitive performance. For example, where choices like sleep duration and alcohol are concerned, one might predict a U-shaped relationship, where too much or too little may be equally bad. In such cases, standard correlational or general linear models may not be appropriate. In addition, random forest regression can perform with high accuracy when there are several unique combinations of factors that can lead to the same outcome. For example, while exercising regularly and eating a vegan diet may both confer benefits for cognition, sleeping eight hours per night and socializing regularly may be equally beneficial.

In the present study, we investigated whether combinations of lifestyle choices can accurately predict whether cognitive performance will be above or below the population mean in over 10,000 participants, using recursive feature elimination (RFE) and random forest regression. We hypothesized that we would be able to predict cognitive scores based on personal combinations of lifestyle choices. Second, we hypothesized that different optimal combinations of choices would apply, depending on the specific cognitive process under investigation; that is, the combination that best predicted performance in one cognitive domain (e.g., short-term memory) would differ from that which best predicted performance in another (e.g., reasoning).

## Materials and methods

### Participants

An international sample of 40,105 participants registered for the online study [10] between June 23, 2017 and February 5, 2018. Participants were only included in the final data analysis if they completed all relevant questionnaire items and all 12 tests, eliminating 23,293 people. Most of this attrition was due to technical issues related to server performance during the initial surge of registrations that prevented participants from completing stages of the experiment. After data cleaning (described below), 9,443 participants (5,954 identified as female, 3,407 identified as male, 82 identified as ‘other’) were included in the final data analysis, ranging in age from 18–69 years (M = 39.67, SD = 13.05). Descriptive information is summarized in Table 1. The experimental protocol was approved by Western University’s Office of Human Research Ethics (protocol ID #109196) and all participants provided informed implied consent by clicking a button to complete the survey prior to participating.

**Table 1:** Demographic summary of participants.

Measure	Value
*n*	9,443
Age (years)	M = 39.67 (SD = 13.05)
Gender	
Female	5,954 (63%)
Male	3,407 (36%)
Other	82 (0.87%)
Socioeconomic status	
At or above the poverty line	8,785 (93%)
Below the poverty line	658 (7%)

### Materials

#### Cognitive tests

Twelve cognitive tests were used to assess a broad range of executive functions, such as inhibition, working memory, problem-solving, and planning. These 12 tests have been validated in patients with anatomically specific frontal-lobe lesions [35, 36], in neurodegenerative populations with frontostriatal cognitive impairments [37], and in pharmacological intervention studies [38]. Functional-neuroimaging studies in healthy adults [39] and in neuropathological populations [40] have shown these tests to be associated with activity in frontal or frontostriatal circuitry. The individual tests are described in detail in the S1 File, and test-retest reliability measures are given in S1 Table. The twelve cognitive tests were used to create three factor scores reflecting performance in three cognitive domains, henceforth referred to as ‘Short-Term Memory’, ‘Reasoning’, and ‘Verbal Ability’, as described previously by Hampshire and colleagues [39]. These 12 tests, their cognitive assessment purposes, and factor scores are summarized in Table 2.

**Table 2:** Cognitive tests and their PCA loadings onto short-term memory, reasoning, and verbal abilities.

Test	Description	PCA loading		
		Short-Term Memory	Reasoning	Verbal Abilities
Spatial Span	Spatial short-term memory	0.69	0.22	-
Grammatical Reasoning	Verbal reasoning	0.69	0.21	-
Double Trouble	Response inhibition	0.62	0.16	0.16
Odd One Out	Deductive reasoning	0.58	-	0.25
Monkey Ladder	Visuospatial working memory	0.41	0.45	-
Rotations	Mental rotation	0.14	0.66	-
Feature Match	Feature-based concentration and attention	0.15	0.57	0.22
Digit Span	Verbal working memory	-	0.54	0.3
Spatial Planning	Executive function and planning	0.19	0.52	-0.14
Paired Associates	Episodic memory	0.26	-0.2	0.71
Polygons	Visuospatial processing	-	0.33	0.66
Token Search	Strategy and working memory	0.22	0.35	0.51

The composite domain scores (or ‘factors’) were calculated as follows. First, the individual test scores were normalized (M = 0.0, SD = 1.0). Then, the domain scores were calculated using the formula Y = X (Ar^+^)^T^, where Y is the N × 3 matrix of domain scores, X is the N × 12 matrix of test *z*-scores, and Ar is the 12 × 3 matrix of Varimax-rotated principal component weights from Hampshire et al. [39] All 12 tests contributed to each domain score, as determined by their component weights.

#### Socio-demographic questionnaire

In order to obtain information about lifestyle choices, as well as pertinent demographic information including socio-economic status (SES), age, and gender, participants completed a detailed socio-demographic questionnaire. Twenty of the items from the 66-item socio-demographic questionnaire were determined to be ‘lifestyle choices’ (operationally defined as an activity or habit that one has some control over, rather than a characteristic such as age that is not a matter of choice), and thus relevant to the current study. These items were as follows: average hours of sleep per night, units of alcohol consumed per week, caffeine per day, number of cigarettes smoked per day, recreational drug use, board game frequency, crossword, sudoku, and other puzzle frequency, frequency of playing card games, frequency of playing video games, brain training participation, highest level of education attained, belief in religion, exercise frequency, meditation frequency, musical instruments currently played, number of languages currently spoken, number of pets, social contact frequency, special diets, and use of supplements specifically marketed as cognition-enhancing (nootropics). The 20 items and their corresponding response choices are included in S2 File.

### Procedure

All data were collected with the Creyos (www.creyos.com) online platform. Recruitment was through advertisements on social media platforms (including Twitter and Facebook) and word of mouth, and participants received no compensation for their participation. Upon accessing the Creyos website and beginning the study process, participants read a letter of information, instructions and a letter of consent, acknowledging their fluency in English. After providing informed consent and an email address, participants completed the detailed socio-demographic and lifestyle questionnaire. Next, participants were asked to complete the 12 tests in the Creyos battery, measuring a range of executive functions, including, but not limited to, working memory, reasoning, problem solving, planning, decision-making and verbal abilities. The order of the 12 tests was randomized across participants. Completing the process of registration, the socio-demographic questionnaire and the 12 tests took approximately 60 minutes.

### Statistical analysis

All code used for cleaning, running analyses, and creating figures is available at https://osf.io/nwbx6/.

#### Data cleaning and reduction

Participants were excluded if they did not complete all 12 tests and all relevant questionnaire items, if they got 0 correct on a test, or if they did not perform above chance on any test. Participants were also excluded from the analysis if they reported their age to be less than 18 or greater than 69, due to low numbers outside those limits. Data were then cleaned to remove impossible and improbable questionnaire responses: individuals who reported sleeping more than 24 hours a day (11 participants), smoking more than 100 cigarettes a day (one participant), consuming more than 100 alcoholic drinks a week (four participants), and owning more than 30 pets (six participants) were removed. Of the 40,105 participants who registered for the study, 9,443 participants were included in the final data analysis.

Statistical analysis was performed in R (version 4.0.3). In order to control for the effects of SES, gender, and age, linear regression was performed on each cognitive domain score with all three variables as regressors, including a quadratic term for age. The residual domain scores resulting from each regression were then carried forward for the remainder of the analysis. For the purposes of applying machine-learning techniques, nominal categorical lifestyle variables were converted to numeric using one-hot encoding (*qdapTools* package, version 1.3.5), creating a binary variable for each level of the factor. That is, each level of the nominal categorical factor became its own variable, with a value of 0 or 1. For example, in the case of number of musical instruments played, there were eight categories, including “none”, and so this variable was coded into eight individual columns. If an individual played woodwind and brass instruments, both of these variables would be coded as 1, while the other instrument variables would be coded as 0. If an individual played no instruments, “none” would be coded as 1, and all other instrument columns would be coded as 0. One-hot encoding also allowed us to accurately handle cases where multiple response items were selected (e.g., a participant who reported playing both string and brass instruments). This resulted in a total of 40 individual lifestyle choices, or “features”.

#### Machine leaning model

Random forest regression models were used to predict composite cognitive scores from the 40 lifestyle features. That is, a separate regression model was constructed for each cognitive score that used that same set of predictors. Model performance was scored during training (including feature selection and model tuning stages) and at the final test stage using root mean square error (RMSE), as it measures, on average, how much the predicted value deviates from the actual value, providing a measure of model fit.

Data were first split into 70% training data (for hyperparameter tuning and feature selection) and 30% test data (used to evaluate model performance at the last stage). Next, feature selection was performed on the training data in order to reduce model complexity and improve the accuracy of the random forest regression. Feature selection is a way of reducing the input features and strengthening the prediction results by including only relevant and meaningful features in the model [41]. Recursive Feature Elimination (RFE) was used to determine the optimal number of lifestyle factors to maximize model fit, due to its ability to handle correlation between predictors [42]. Briefly, RFE starts by fitting and scoring the random forest regression, ranking the features by permutation importance, discarding the least important feature, and repeating this process until only one feature remains. Feature ranking was performed using permutation importance, which considers a variable important if it has a positive effect on the prediction accuracy. At each stage (i.e., for a given set of features) the cross-validated score (RMSE) is collected, so that the set of features yielding the best score can be selected for final model evaluation. RFE was performed separately for each of the three regression models (one for each cognitive score) using the rfe function in the *caret* package (version 6.0), and each set of features was evaluated with 5-fold cross-validation. To assess similarity between feature rankings, a Kendall’s Rank Correlation Test was used.

The random forest method has several parameters that can impact model performance (i.e., hyperparameters), and their optimal values can differ dramatically between different kinds of datasets. We selected the hyperparameter values for our models using a grid search, in which all combinations of pre-selected hyperparameter values were used train the three random forest models and evaluate their performance. The random forest regression model was trained with 500 trees in order to reduce variance in the model while maintaining computational efficiency.

Finally, we measured the regression models’ performance on the left-out test dataset (N = 2,833). For each cognitive score, a random forest regression model was trained on the entire training dataset using the hyperparameters and features selected during model tuning, and scored on the test dataset by calculating RMSE and the proportion of variance explained (R^2^). Feature importance was similarly assessed using permutation importance. In order to avoid biasing the results based on the initial 70/30 split, this entire procedure was conducted 100 times, and the mean RMSE and R^2^ values, feature importances, and optimization parameters were calculated.

## Results

### Feature selection and tuning

All reported values represent the mean of 100 iterations. For Short-Term Memory, the average lowest cross-validated RMSE was 1.86, with a mean of 37 features being selected. For Reasoning, the average lowest cross-validated RMSE was 1.70, with a mean of 37 features being selected. For Verbal Abilities, the average lowest cross-validated RMSE was 1.33, with a mean of 37 features being selected.

The set of hyperparameters that produced the best root-mean-square error was selected for each model. This grid search was performed on the training data using the *ranger* package (version 0.13.1). For Short-Term Memory, RMSE was lowest with a mean mtry parameter (the number of variables to randomly sample as candidates at each split) of 9 and a mean minimum node size of 110. For Reasoning, RMSE was lowest with a mean mtry parameter of 9 and a mean minimum node size of 128. For Verbal Abilities, RMSE was lowest with a mean mtry parameter of 11 and a mean minimum node size of 129.

Kendall’s Rank Correlation Tests revealed that ranking of each item was statistically correlated between each domain (Short-Term Memory and Reasoning: tau = 0.72, *p* < .001; Short-Term Memory and Verbal Abilities: tau = 0.73, *p* < .001; Reasoning and Verbal Abilities: tau = 0.69, *p* < .001).

### Model performance

Breakdown of model performance metrics for all three cognitive domains is shown in Table 3. The optimized random forest regression was able to explain 9.3% of the variance in Short-Term Memory scores in the training data. In the case of Reasoning, the regression model explained 7.8% of the variance in scores. In terms of Verbal Abilities, the regression model explained 6.7% of variance in scores.

**Table 3:** Final regression output for short-term memory, reasoning, and verbal ability classes.

Cognitive domain	R^2^	Root-mean-square error
Short-Term Memory	0.09	1.85
Reasoning	0.08	1.69
Verbal Abilities	0.07	1.32

### Feature importance

**Fig 1:**
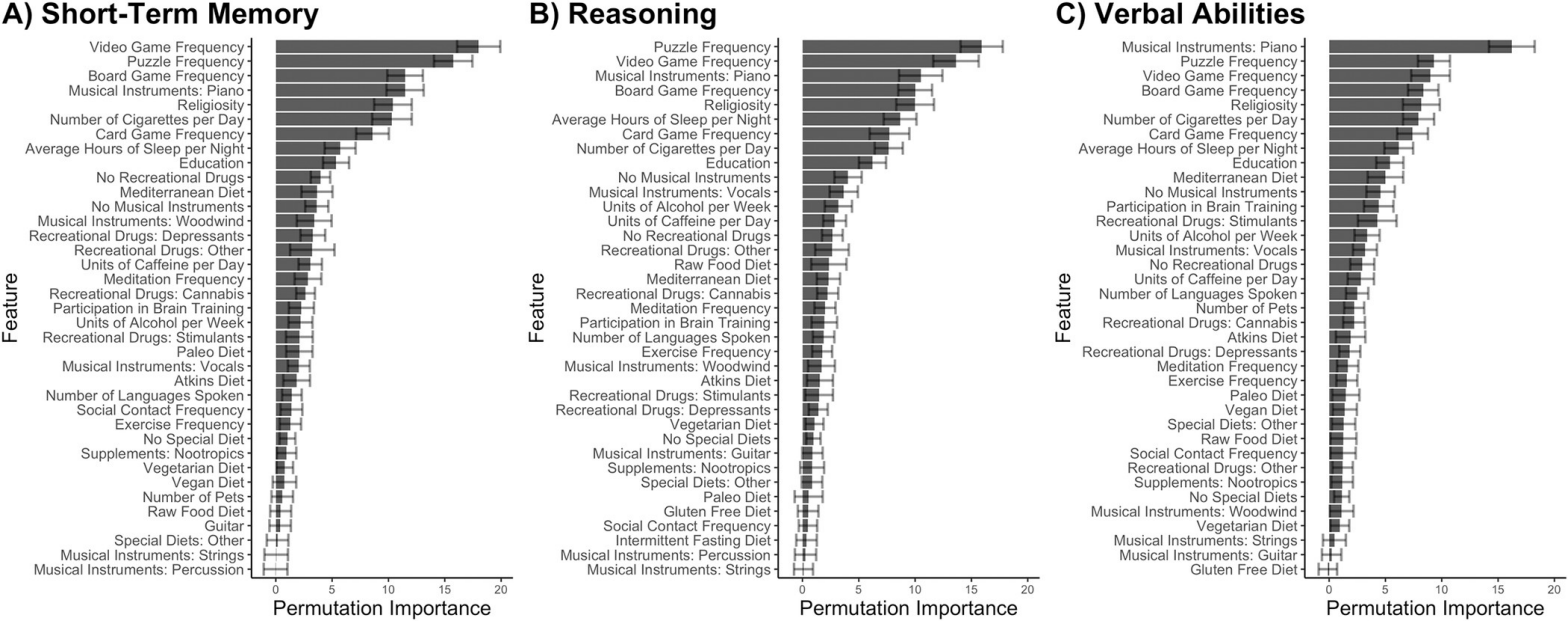
Feature ranking for A) Short-Term Memory, B) Reasoning, and C) Verbal Abilities. Features are ranked by permutation importance. A value of 0 means that a feature has no effect on prediction accuracy.

**Fig 2:**
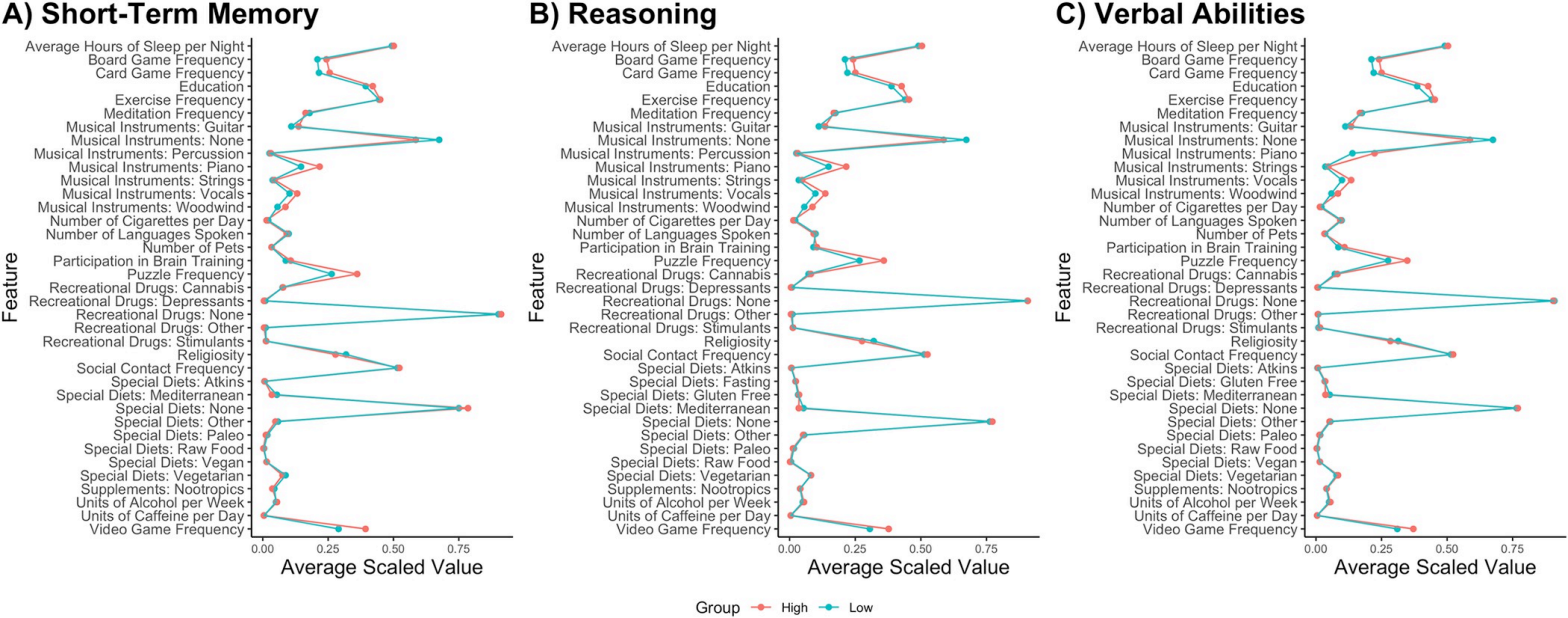
Mean responses of features that provided the most information gain, averaged for individuals who performed above and below the mean, for A) Short-Term Memory, B) Reasoning, and C) Verbal Abilities. All features were scaled to a range of 0–1.

Rankings for each final random forest regression model are shown in Fig 1. The top three features that provided information to the Short-Term Memory regression were frequency of playing video games, frequency of doing puzzles, and frequency of playing board games. The top three features that provided information to the Reasoning regression were frequency of doing puzzles, frequency of playing video games, and playing the piano. The top three features that provided information to the Verbal Ability regression were playing the piano, frequency of doing puzzles, and frequency of playing video games. In all three cognitive domains, the rest of the predictors that were retained pertained to playing instruments, religious involvement, education level, exercise, sleep, diet, and other hobbies such as playing cards and board games. Averages for each feature across the entire sample are shown in Fig 2.

## Discussion

In this study of 9,443 participants, the relationship between lifestyle choices and cognitive abilities was examined using machine learning techniques. In an international sample, we were able to explain 7–9% of the variance in cognitive scores in Short-Term Memory, Reasoning, and Verbal Abilities, based on 20 lifestyle choices. Second, we found unique rankings of predictors for each cognitive domain, although there was statistically significant overlap in the features that contributed most to each of these models, suggesting that in general there are some commonalities among those lifestyle choices that tend to influence cognitive function most.

In all three cognitive domains, the frequency with which an individual played video games appeared among the most important features for predicting performance. The relationship between video games and cognitive outcomes is contentious within the literature; there is a wealth of research that has demonstrated that video game players outperform their counterparts on tests of working memory, attention, executive control and visuo-spatial abilities [19, 43–45]. Researchers have proposed that these cognitive gains are thought to be due to enhanced attentional control, evidenced by differences in attention-linked parietal function [46], event-related potentials [47, 48], and steady state visual evoked potentials [49, 50] between different kinds of gamers as well as non-gamers. In order to determine causality, that is, whether these differences are a result of neuroplasticity within these regions due to playing video games, or represent pre-existing individual variability that leads to an increased interest in gaming, intervention studies are required. Results of such studies, in which non-gamers have been trained on specific video games, have shown both structural and functional brain changes [51–53], although the location of these effects has differed. However, several other studies have failed to find cognitive differences, failed to replicate previous positive results, and have suggested that the improvements in video game playing do not extend beyond the game itself [21, 23, 54]. Some of these discrepancies may occur not only because of the type of video game being studied [55], but also because in the literature, video game playing is largely considered in isolation rather than in the context of the overall set of lifestyle choices that might accompany it and exert an influence. For example, in the current study, video game playing did not singularly predict cognitive ability; rather, factors such as sleep, education, and religiosity contributed predictive power as well. This suggests that any study examining the influence of video game playing on cognitive ability should, at the very least, take account of these additional contributory factors. In very simple terms, one can easily imagine an interaction between video game playing and sleep, where the potential beneficial influence of the former might be counteracted by its detrimental influence on the latter.

Although video gaming emerged as being among the most important features for predicting cognitive ability, we found that the frequency of solving puzzles and playing card/board games also provided predictive information for cognitive performance. Engaging in these activities was associated with above average performance (see Fig 2), suggesting that there may be a common factor present in all four hobbies that may underly the relationship with cognition. For example, factors such as social interaction, reward, and strategic thinking are common to all of these activities. Again, this confirms that lifestyle choices such as video game playing should not be considered in isolation when exploring the potential effects on cognition because it may simply reflect a more general factor inherent in such hobbies.

Several themes emerged amongst the features ranked as most important in all three cognitive domains. For example, playing a musical instrument was important for predicting Short-Term Memory, Reasoning, and Verbal abilities, with those performing above the mean being more likely to report playing an instrument in all three cases (Fig 2). Another was an individual’s belief in religion, with participants who performed cognitively above average reporting less religious involvement in all three domains. Unsurprisingly perhaps, education level also appeared as an important feature in all three domains.

As we predicted, although there was significant overlap in the features that contributed to each cognitive domain, the overall set and ranking of features was unique in each case. This suggests that whilst making informed lifestyle choices can be beneficial for cognition, the relationship between the two is somewhat nuanced. For example, while number of pets contributed to predicting Short-Term Memory and Verbal Abilities, it did not contribute to Reasoning at all. In contrast, whether or not someone followed an intermittent fasting diet contributed somewhat to Reasoning ability, and not at all to Short-Term Memory or Verbal Abilities. This supports the view that human cognition is not unitary, but, rather, is formed from multiple components organized into functionally specialized networks [39]. Indeed, the same three cognitive domains examined in this study (Short-Term Memory, Reasoning, and Verbal Abilities) have been shown to recruit specific and dissociable neuroanatomical networks in the human brain [39]. Thus while some lifestyle factors may be globally beneficial to cognition, it is unsurprising that some affect specific domains more than others.

Importantly, based on the results of the present study, no conclusions can be drawn about causation. That is, while some of the lifestyle factors examined may affect cognition, others may themselves be affected *by* cognition. For example, while board games may improve short-term memory and reasoning by teaching these skills, it is equally likely that people with better short-term memory and reasoning abilities enjoy playing board games because they are good at them, and thus play them more often. This conflation of correlation with causality is one factor that has led to the considerable public enthusiasm for, and commercial investment in, “brain training” interventions, despite there being little evidence to support their worth [29, 30]. Additionally, despite the large sample size, there was a high rate of data loss due to server issues and strict data cleaning methods, which could impact the generalizability of the study. Finally, we did not screen participants for cognitive impairments such as dementia. Although the data were cleaned to exclude anyone who scored 0 or performed below chance on any test as well as outliers, it is possible that the data includes individuals with cognitive impairment.

Our regression models explained approximately 7–9% of variance in cognitive scores. While our models provided predictive value, this large set of lifestyle features still leaves a considerable amount of residual variance unaccounted for, even after controlling for age, gender, and SES. This result again has implications for interventions aimed at improving cognitive function, or preventing decline, because it suggests that while it is likely that gains can be made, there are clear limits on what can be achieved.

We conclude by emphasizing that, while the relationship between lifestyle choices and cognition is important, it is far from straightforward. Undoubtedly, the choices we make in our lives are associated with our cognitive abilities, and thus may influence our daily functioning and promote healthy aging [56]. Nevertheless, there is no one-to-one mapping between any particular lifestyle choice and improved cognition, suggesting that there is no universal optimal design for life.

## Supporting information

S1 Table Test-retest reliability of the 12 cognitive tasks. (DOCX)

S1 File Test descriptions. (DOCX)

S2 File Questions from the socio-demographic questionnaire included in the present study. (DOCX)
