# Supplementary material for: A design for life: Predicting cognitive performance from lifestyle choices
Source: PLoS One. 2024 Apr 16;19(4):e0298899. doi: 10.1371/journal.pone.0298899 (PMC11020841; doi:10.1371/journal.pone.0298899)
Supplement: S1 Table — (DOCX) [file pone.0298899.s001.docx]

| **Table S1.** **Test-retest reliability of the 12 cognitive tasks** | |
| --- | --- |
| Task | Pearson’s *r* |
| Double Trouble | 0.82 |
| Spatial Planning | 0.73 |
| Odd One Out | 0.47 |
| Grammatical Reasoning | 0.76 |
| Feature Match | 0.61 |
| Polygons | 0.52 |
| Digit Span | 0.62 |
| Rotations | 0.62 |
| Token Search | 0.61 |
| Paired Associates | 0.44 |
| Spatial Span | 0.58 |
| Monkey Ladder | 0.56 |
