# Supplementary material for: A design for life: Predicting cognitive performance from lifestyle choices
Source: PLoS One. 2024 Apr 16;19(4):e0298899. doi: 10.1371/journal.pone.0298899 (PMC11020841; doi:10.1371/journal.pone.0298899)
Supplement: S2 File — (DOCX) [file pone.0298899.s003.docx]

**Supplemental File S2.**

**Questions from the socio-demographic questionnaire included in the present study**

1. What is your date of birth?

- Select one: (Month) Jan, Feb, Mar, Apr, May, June, July, Aug, Sept, Oct, Nov, Dec
- Select one: (Year) 1900-2017

1. What is your gender?

- Select one: Female, Male, Non-binary/third gender, Prefer not to say

1. How would you describe the economic status of your family when you were growing up?

- Select one: At or above poverty level, below poverty level

1. How many hours of sleep do you normally get per day?
   - Select one: 0-24
2. How many languages do you speak?
   - Select one: 1-20
3. What is your level of education?

- Select one:
  - No certificate, diploma, or degree
  - High school diploma or equivalent
  - Some university/college, no diploma
  - College diploma
  - Undergraduate degree
  - Graduate degree

1. How would you rate your belief in religion?
   - Select multiple: Atheist, Agnostic, Religious lapsed, Religious practicing, Very religious
2. Do you participate in brain training programs?
   - Select one: Yes, No
3. How often do you play video games?
   - Select one: Every day, several times a week, weekly, Infrequently, Never
4. How often do you play card games?
   - Select one: Every day, several times a week, weekly, Infrequently, Never
5. How often do you play board games?
   - Select one: Every day, several times a week, weekly, Infrequently, Never
6. How often do you do crosswords, Sudoku, or other types of puzzles?
   - Select one: Every day, several times a week, weekly, Infrequently, Never
7. How often do you meditate?
   - Select one: Every day, several times a week, weekly, infrequently, never
8. Which musical instruments do you play?
   - Select multiple:
     - Piano
     - Vocals
     - Guitar (electric, acoustic, bass)
     - Strings (violin, cello, etc.)
     - Percussion
     - Brass (trumpet, trombone, tuba, etc.)
     - Woodwind (clarinet, saxophone, etc.)
9. How many cigarettes do you smoke each day?
   - Select one: 0-100
10. How many units of alcohol do you drink each week? (1 unit = 1 beer, 1 glass of wine, 1oz spirits)
    - Select one: 0-100
11. How many caffeine containing drinks do you consume each day?
    - Select one: 0-100
12. Have you ever taken any drugs recreationally?
    - Select multiple:
      - Cannabis
      - Stimulants (amphetamines, cocaine, etc.)
      - Depressants (Opioids and opiates, barbiturates, tranquilizers, etc.)
      - Other
13. Do you take supplements or drugs (e.g., nootropics) to enhance your cognition?
    - Select one: Yes (text input), No
14. What special diets do you follow?
    - Select Multiple:
      - Vegetarian
      - Vegan
      - Gluten Free
      - Paleo
      - Atkins
      - Mediterranean
      - Raw Food
      - Fasting
      - Other
      - None
15. How many pets do you have?
    - Select one: 0-100
16. How often do you exercise, such that you work up a sweat?
    - Select one: Every day, Several times a week, Weekly, Infrequently, Never
17. How often do you have social contact with friends or family that you do not live or work with?
    - Select one: Every day, Several times a week, Weekly, Infrequently, Never
